# Supplementary material for: Polyphenol-Rich Liupao Tea Extract Prevents High-Fat Diet-Induced MAFLD by Modulating the Gut Microbiota
Source: Nutrients. 2022 Nov 21;14(22):4930. doi: 10.3390/nu14224930 (PMC9697786; doi:10.3390/nu14224930)
Supplement: Supplementary file 1 [file nutrients-14-04930-s001.zip › nutrients-1942765-supplementary.pdf]

Supplementary Materials

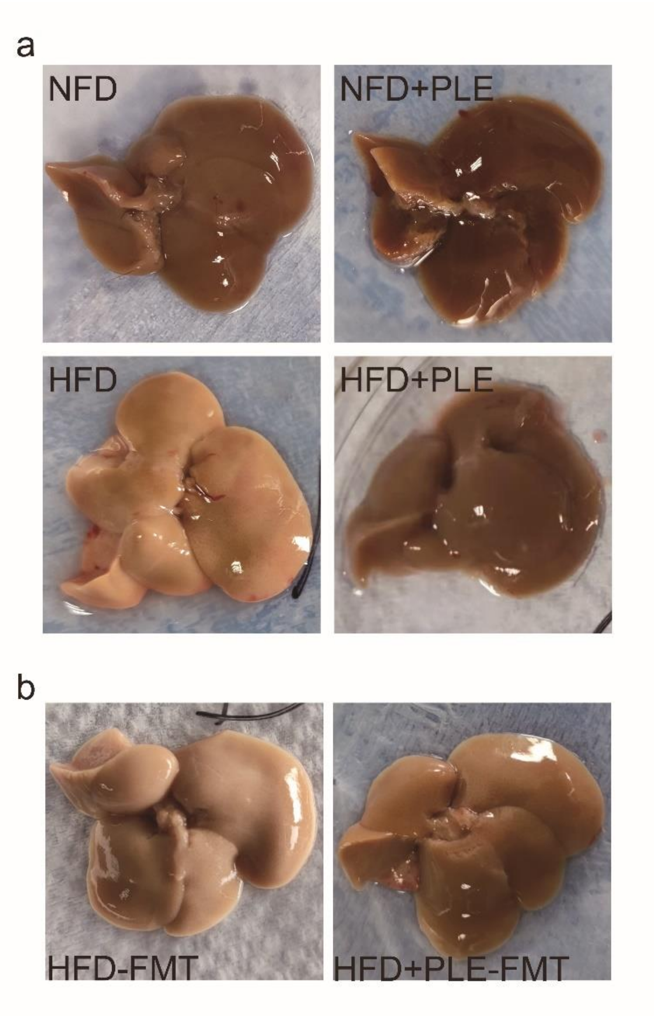

Figure S1. Visual observation of the liver of mice in each group.

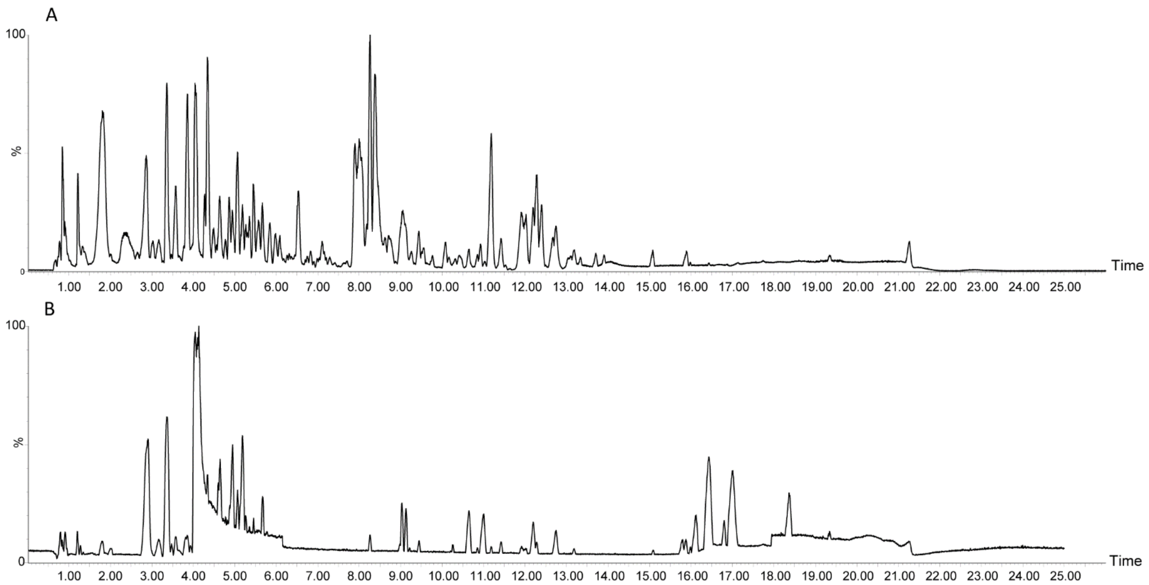

Figure S2. Ultra-high-performance liquid chromatography with quadrupole time-of-flight mass spectrometry (UPLC-QTOF/MS) chromatograms of polyphenol-rich Liupao Tea extract (PLE). A: Negative ion mode; B: Positive ion mode.

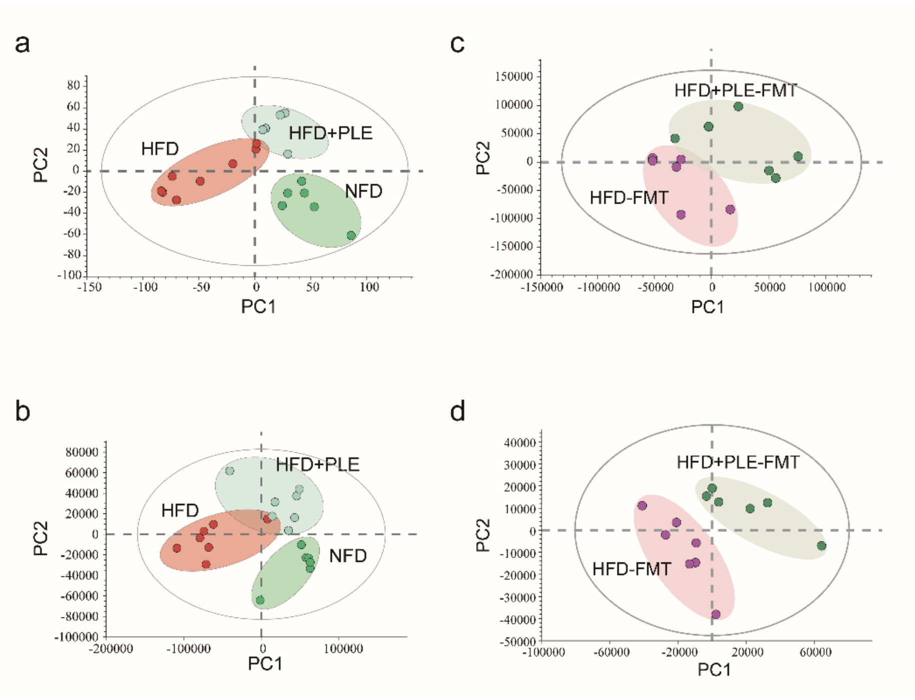

Figure S3. PLS-DA score plots based on the UHPLC/Q-TOF MS spectra in each group.

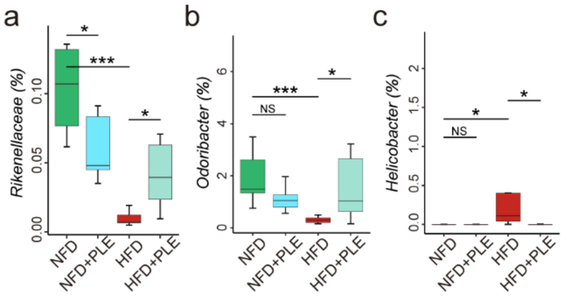

Figure S4. Abundance of the *Rikenellaceae*, *Odoribacter*, and *Helicobacter* genera in the intestinal tract of mice in each group.
